# Single-cell epigenetics and multiomics analysis in kidney research

**DOI:** 10.1007/s10157-025-02679-8

**Published:** 2025-04-25

**Authors:** Seishi Aihara, Yoshiharu Muto

**Affiliations:** Division of Nephrology, Department of Internal Medicine, University of Texas Southwestern Medical Center, 5901 Forest Park Rd., Dallas, TX 75390 USA

**Keywords:** Single-cell analysis, Epigenetics, Multiomics, Kidney

## Abstract

The rapid evolution of single-cell sequencing technologies has significantly advanced our knowledge of cellular heterogeneity and the underlying molecular basis in healthy and diseased kidneys. While single-cell transcriptomic analysis excels in characterizing cell states in the heterogeneous population, the complex regulatory mechanisms governing the gene expressions are difficult to decipher using transcriptomic data alone. Single-cell sequencing technology has recently extended to include epigenome and other modalities, allowing single-cell multiomics analysis. Especially, the integrative analysis of epigenome and transcriptome dissects the cell-specific, gene-regulatory mechanisms driving cellular heterogeneity. An increasing number of single-cell multimodal atlases are being generated in nephrology research, offering novel insights into cellular diversity and the underpinning epigenetic regulation. This ongoing paradigm shift in kidney research accelerates the identification of new biomarkers and potential therapeutic targets, promoting clinical translation. In this era of transformative nephrology research, the basic knowledge of single-cell sequencing analysis and multiomics approach is valuable not only for basic science researchers but for all nephrologists. This review overview single-cell analysis, with a focus on emerging epigenomic and multiomics approaches and their application to kidney research.

## Introduction

The kidney is composed of diverse cell types and subpopulations organized within the complex structures. This cellular heterogeneity has hampered our efforts to understand molecular physiology and pathophysiology of the kidney. Single-cell sequencing analysis is an emerging research strategy designed to quantify a biological property such as transcriptome at a single-cell resolution. Simultaneous measurements for tens of thousands of cells unravel cellular heterogeneity [1], allowing the comprehensive molecular understanding of cell states. Over the past decade, single-cell sequencing technology and analysis have advanced rapidly, driven by interdisciplinary collaborations of molecular biology, chemistry, fluidics, electronics and computer science. Following the maturation of single-cell transcriptomics approaches, single-cell sequencing approach has recently extended to include epigenomics as well as other omics methodologies, even multimodal analysis at a single-cell resolution [2]. This facilitates our understanding of genome-wide gene regulation beyond the transcriptome as well as the identification of new biomarkers and potential therapeutic targets, accelerating clinical translation. Furthermore, the rapid innovation of single-cell sequencing technology has driven significant improvement in computational analysis methods. Subsequent development of numerous user-friendly and efficient computational tools has enabled any researcher to start single-cell analysis without expertise in computational skills. In the middle of the paradigm shift in kidney research that may be rapidly translated to clinical medicine, we believe that basic knowledge of single-cell sequencing analysis—particularly multiomics approaches beyond the transcriptome—is valuable not only for basic researchers but for all nephrologists. This review focuses on single-cell analysis with emphasis on epigenomic analysis and multiomics approaches, aiming to provide a clear and comprehensive overview for researchers and clinicians involved in nephrology.

## Review

### Single-cell transcriptomics

Traditional research approaches to the kidney and other organs frequently generate data that represent mixed signals from multiple cell types, obscuring the contributions of individual populations. For example, typical bulk RNA sequencing (RNA-seq) of kidney samples measures mRNA levels from homogenized tissue including epithelia from multiple compartments as well as interstitium, thereby masking signals from rare cell types [3]. Moreover, the mixing of signals from multiple cell types hinders our ability to understand intercellular communications and cell-type-specific gene regulatory networks. Single-cell analysis is a research strategy to overcome these limitations of bulk-profiling methodologies. When sufficient cells are included in the single-cell dataset, subsequent computational analysis recovers low-abundance cell types that are often challenging to capture by conventional experimental methodologies. Additionally, the computational analysis leveraged by machine learning allows the modeling of cell lineage dynamics [4], prediction of cell-to-cell interactions [5] and even inference of gene regulatory network [6].

The first widespread application of single-cell sequencing analysis focused on the transcriptome. Following the advent of single-cell RNA sequencing (scRNA-seq) in 2009[7], a cascade of experimental innovations has occurred, each accompanied by continuous refinement. This rapid technological evolution has promoted parallel improvement of computational analysis methods, culminating in the development of over a thousand tools and software packages [8]. Platforms such as Bioconductor [9], along with user-friendly tools like Seurat [10] and well-documented pipelines, benchmarks, and best-practice workflows [8], have made scRNA-seq analysis accessible to all the researchers including ones without substantial expertise in computational analysis, driving biological discoveries in all the biomedical research fields including nephrology. Single-cell transcriptomic analysis has been applied to both human and mouse kidneys, fundamentally improving our understanding of cellular heterogeneity within the kidney [11]. The increasing number of transcriptomic atlases has brought critical insights into molecular nephrology, facilitating the identification of potential biomarkers and therapeutic targets.

### Single-cell vs single-nucleus analysis

Before diving into a discussion of the details of single-cell sequencing analysis, we might want to clarify the definitions of two terms: single-*cell* and single-*nucleus* analysis. The initial step in a single-cell analysis experiment involves tissue dissociation. However, isolating single cells from the human kidney and other solid organs poses significant challenges, as these tissues typically require intensive mechanical and enzymatic dissociation. Such procedures often fail to capture all kidney cell types accurately, as the dissociation process can damage fragile cells and fail to release cells embedded within the extracellular matrix. Moreover, this procedure can introduce gene expression changes by triggering various cellular stress responses to mechanical and enzymatic dissociation [11–13].

An alternative approach to circumvent these limitations is a single-nucleus approach. This method, widely adopted in kidney researches, involves isolating nuclei through dissociation of frozen tissue and subsequent cell lysis, utilizing single-nucleus suspensions for downstream library preparation and sequencing analysis. This single-nucleus RNA sequencing (snRNA-seq) analysis is capable of detecting a broader range of cell types, including podocytes and other glomerular cells, which are often underrepresented in single-cell suspension [12]. Another advantage of snRNA-seq is its compatibility with frozen samples, promoting the utilization of cryopreserved human kidney samples. The details of biological differences between snRNA-seq and scRNA-seq are not discussed here, as they are reviewed elsewhere [11, 12].

A key limitation of snRNA-seq is its inability to sequence mitochondrial reads, which underrepresents molecular changes in mitochondrial pathways [14]. Recently modified protocols of single-cell sequencing techniques have enabled profiling mitochondrial genome information such as copy number and mutations [15–17], allowing for mitochondrial analysis and lineage tracing. Given that most of the kidney diseases are associated with mitochondrial dysfunction [18–20], the application of mitochondrial sequencing to nephrology research will enhance our understanding of mitochondrial pathways altered in kidney diseases.

It is also important to note that “single-cell” epigenetics analysis focuses on the nuclear content, requiring typically single-nucleus isolation. In addition, joint profiling of “single-cell” transcriptome and epigenome usually needs nuclei isolation from the tissues. For simplicity, both single-*cell* and single-*nucleus* methods will be collectively referred to as single-cell analysis throughout this review.

### Single-cell epigenomics

While scRNA-seq excels in characterizing cellular heterogeneity and describing individual cell states, it offers only a snapshot of a dynamic biological process — The complex regulatory mechanisms underlying the transcriptome are difficult to decipher using scRNA-seq data alone. Transcriptional regulation is orchestrated through complicated interactions among genes, chromatin, transcription factors and cofactors, collectively forming gene regulatory network [21]. To better elucidate the molecular basis that define cell states, researchers have devoted significant effort to developing methods capable of measuring epigenetic modalities at single-cell resolution [2]. Such epigenetic modalities include chromatin accessibility, histone modifications, and DNA methylation. Among these, chromatin accessibility profiling is currently the most mature and commercially available approach for single-cell, genome-wide epigenetic studies.

Chromatin is a complex structure composed of DNA and DNA-associated proteins, including histones, transcription factors, cofactors and other proteins such as a chromatin remodeling complex [22]. In eukaryotic cells, the majority of genomic DNA is organized into nucleosomes, with each nucleosome consisting of approximately 146 base pairs of DNA wrapped around a histone octamer [22]. Chromatin accessibility refers to the extent to which macromolecules, such as proteins, can physically access genomic DNA within the chromatin [23]. This accessibility is determined by the level of chromatin compaction, which is mainly dependent on the occupancy of nucleosomes. Of note, chromatin accessibility is dynamic and uneven across the genome and also often cell-type-specific [23]. Functional genomic regions, such as active enhancers and promoters, tend to have less frequent nucleosomes and greater accessibility for DNA-binding proteins, enabling precise transcriptional regulation. Therefore, chromatin accessibility profiling allows us to learn the genome-wide functional genomic elements involved in transcriptional regulation.

ATAC-seq (Assay for Transposase-Accessible Chromatin using sequencing) is a widely used technique for identifying genome-wide open chromatin regions [24]. This method employs the hyperactive Tn5 transposase enzyme preloaded with sequencing adapters. When the Tn5 transposase accesses genomic DNA in an open chromatin region, it simultaneously integrates sequencing adapters and cuts the DNA. This process generates numerous DNA fragments derived from accessible chromatin regions across the genome. By sequencing these fragments, the genome-wide open chromatin regions are determined. ATAC-seq has gained popularity due to its low cell input requirement (fewer than 50,000 cells) and relatively simple protocol, which circumvent the limitations of other chromatin accessibility profiling methods, such as DNase-seq and MNase-seq [24].

**Fig. 1:**
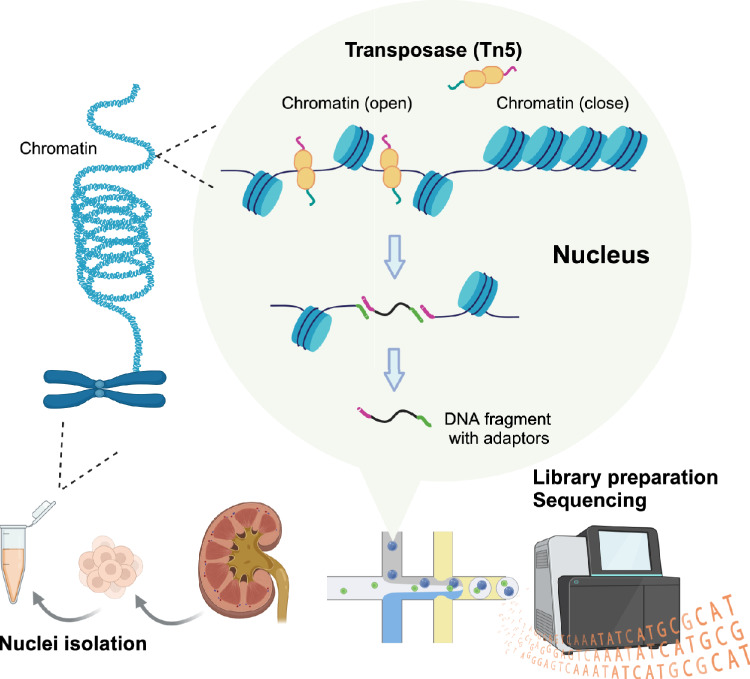
Scheme of single-cell ATAC-seq. Single-cell ATAC-seq (scATAC-seq) allows profiling of chromatin accessibility at the single-cell resolution. Following nuclei extraction from a frozen sample, the nuclei are subjected to a transposition reaction, during which the Tn5 transposase inserts sequencing adapters and cuts the accessible regions in each nucleus. The transposed DNA fragments in each nucleus are further labeled with a unique, cell-specific DNA barcode, then amplified to prepare a sequencing library. Created with Biorender.com

Single-cell ATAC-seq (scATAC-seq) is an extension of ATAC-seq to single-cell analysis, profiling chromatin accessibility at the single-cell resolution (Fig. 1) [25]. The scATAC-seq protocol typically begins with the isolation of nuclei from a frozen biosample [11]. The nuclei are then subjected to a transposition reaction, during which the Tn5 transposase inserts sequencing adapters into open chromatin regions of individual nuclei. Subsequently, individual nuclei are isolated using methods such as programmable microfluidic platform [25], combinatorial indexing [26], or droplet microfluidics [27]. The transposed DNA fragments in each nucleus are further labeled with a unique DNA barcode to distinguish them from fragments originating from other nuclei. The barcoded fragments are then amplified to prepare a sequencing library, producing millions of short DNA reads that correspond to regions of open chromatin in each nucleus. Among the single-cell isolation strategies, a droplet-based method was commercialized first [27] and most widely used in single-cell epigenomics.

### Data analysis in single-cell chromatin accessibility profiling

**Fig. 2:**
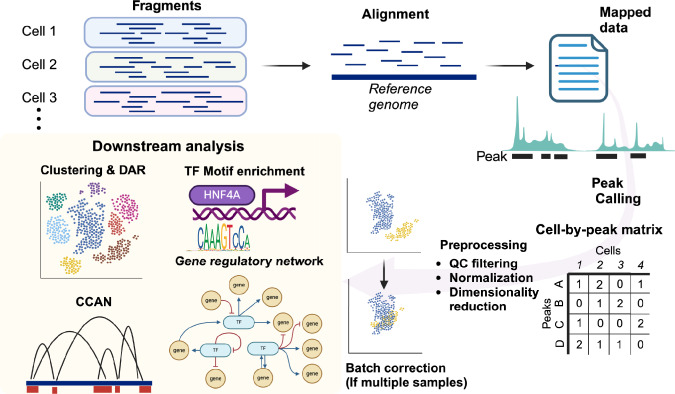
Preprocessing and basic analyses for scATAC-seq data. Preprocessing workflow for scATAC-seq involves alignment of the fragments to reference genome, peak call of the fragments to generate a cell-by-peak matrix and preprocessing including quality control, normalization and dimensionality reduction. The batch differences among samples may be corrected by another normalization methods. Subsequent secondary analyses including but not limited to the identification of differentially accessible regions (DAR) among the clusters, transcription factor (TF) motif enrichment analysis, cis-coaccessibility network (CCAN) analysis and construction of gene regulatory network. Created with Biorender.com

We will overview the data processing in the scATAC-seq to understand how single-cell epigenetic data can bring valuable biological insights (Fig. 2). After sequencing a scATAC-seq library, the first step in the data analysis involves aligning each read to the reference genome assembly and constructing data matrix. There are fundamental differences between scATAC-seq and scRNA-seq data matrices. The scRNA-seq data matrix is defined by gene names as features (rows) and cell barcodes (columns), with the values at each intersection of a row and column representing gene expression level. In contrast, a scATAC-seq data matrix lacks a predefined set of features due to the continuous, genome-wide nature of chromatin accessibility measurements. The most common scATAC-seq data matrix is cell-by-peak matrices [28]. The peaks refer to the genomic regions with high fragment density, indicating open chromatin regions. These regions are identified through a computational process called peak calling [28, 29]. The number of features (typically peaks) in a scATAC-seq matrix is substantially larger than that in a scRNA-seq matrix. In addition to differences in quality and quantities of the features, scATAC-seq matrices are characterized by significant sparsity, as only a small fraction from just two copies of genomic regions is accessible in each cell. These distinct attributes of scATAC-seq data necessitate data analysis strategies that differ substantially from those used for scRNA-seq [3, 8, 28].

Following the generation of the data matrix, the preprocessing steps have been streamlined by established computational tools, such as Seurat [10], Signac [30], ArchR [31], SnapATAC [32] and Cicero [33]. These bioinformatics packages provide standardized frameworks for processing and analyzing scATAC-seq data. Following the removal of low-quality cells based on the quality control metrics, the remaining steps of data preprocessing involve normalization and dimensionality reduction. To address the sparsity of scATAC-seq data, many analysis pipelines like Seurat, Signac and ArchR adopt latent semantic indexing [10, 28, 30, 31], an approach originally developed for natural language processing to evaluate document similarity based on word counts. Other dimensionality reduction strategies include latent Dirichlet allocation [34] and spectral embedding [32]. When multiple datasets need to be compared, additional normalization steps may be required to further correct technical and biological batch effects [35]. These normalizations and dimensionality reduction allow clustering and visualization of the scATAC-seq data.

The clusters in scATAC-seq data are annotated based on differentially accessible regions (DARs) and gene activity. DARs are determined as either cell-type-specific or disease-specific accessible regions by statistical methods. DARs can be linked to nearby genes, providing insights into the cell identity or cell state. Gene activity is a computational prediction of gene expression based on chromatin accessibility on the gene body and surrounding regulatory regions. The open chromatin region identified in scATAC-seq is valuable for understanding the transcriptional regulation of each gene. However, the primary strength of scATAC-seq lies in its ability to provide a comprehensive picture of the genome-wide epigenetic state.

One of the key approaches to interpreting these genome-wide open chromatin regions is motif enrichment analysis [36–38], which quantifies the occurrence of transcription factor binding motifs within all these open chromatin regions or DARs in each cell type. The underlying assumption is that open chromatin regions are either bound or potentially bound by transcription factors whose motifs are present in those regions. The motif enrichment analysis allows inference of the active transcriptional regulators that define cell-type-specific gene expression landscape [38]. Furthermore, correlation in chromatin accessibility between two genomic regions (co-accessibility) in scATAC-seq data is also utilized for inference of cell-specific network of functional genomic elements interacting with each other (cis-coaccessibility network) [33]. The simultaneously accessible regions in a given cell (co-accessible regions) tends to cluster on the genomic DNA, where enhancer and promoters interact physically via chromatin looping, regulating transcription in a coordinated manner. Cis-coaccessibility network identified in scATAC-seq data and useful to identify cell-type-specific enhancer as well as to infer critical transcription factors that bind and regulate these regions [33]. Furthermore, the scATAC-seq data along with scRNA-seq data can be utilized for gene regulatory network analysis [21].

### Single-cell multiomics analysis

**Fig. 3:**
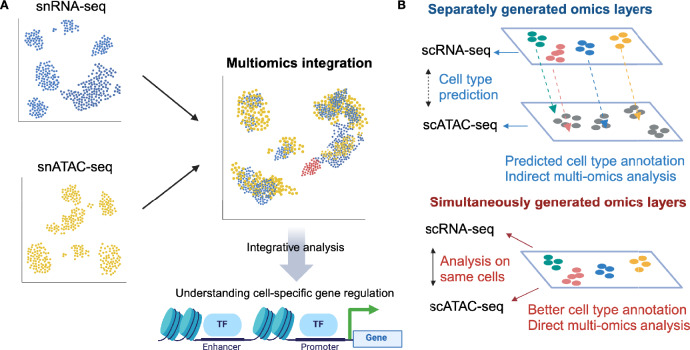
Multimodal integration of single-cell omics data. **A** Integrative analysis of scRNA-seq and scATAC-seq data brings deeper insight into the cells-specific gene regulation landscape, compared to single-omics analysis alone. **B** There are two types of multimodal integration: Integration of unmatched (separately generated) multiomics datasets generated from different cells. Integration of matched (simultaneously generated) multiomics datasets generated from identical cells, allowing enhancement of annotation accuracy and higher-resolution analyses of multiomics analysis. Created with Biorender.com

While each single-cell omics modality contributes to defining the cell states, a multimodal approach allows a deeper and more comprehensive understanding of the biological processes. Especially, the combination of epigenome and transcriptome dissects the mechanism of cell-specific gene regulation driving cellular heterogeneity (Fig. 3A). We have generated separately scRNA-seq and scATAC-seq data on the same human kidney cortex samples [39] and integrated these datasets by anchor-based label transfer protocol [10]. This integration strategy is based on identifying pairs of anchor cells, whose gene expression profiles in scRNA-seq and gene activity profiles computed in scATAC-seq are similar. Those anchors link each cluster between the multimodal datasets and enable comparative analysis between modalities. it is noteworthy to mention that there have been more sophisticated strategies recently developed for unmatched multimodal integration (Fig. 3B) such as graph-linked unified embedding (GLUE) [40] and bridging integration adopted in Seurat v5 [41]. Our analysis characterized chromatin accessibility in the VCAM1-expressing proximal tubule cell (PTC) subset, which we and others have identified through scRNA-seq [39, 42, 43]. This PTC subpopulation shows pro-inflammatory, pro-fibrotic gene expression signature, and their frequency increases in various kidney disease [42, 44, 45], implicated in CKD progression. Motif enrichment and cis-coaccessibility analysis in scATAC-seq combined with gene expression profiling revealed that NF-κB transcription factors are specifically activated in the VCAM1-expressing PTC subset [39], suggesting its potential therapeutic applicability.

Recent technological advances have enabled even simultaneous, matched profiling of scRNA-seq and scATAC-seq within the same single cells (Fig. 3B), allowing enhancement of annotation accuracy and higher-resolution analyses of cell-specific regulatory mechanisms. Ledru et al. applied joint single-cell profiling of transcriptome and chromatin accessibility to human kidney samples [46]. Using a regularized regression strategy to infer genome-wide gene regulatory networks, their approach effectively leveraged multimodal datasets to predict functional genomic elements and transcription factors associated with successful and failed epithelial repair.

### Application of single-cell epigenomics and multiomics to the kidney

The single-cell multiomics approaches are being increasingly applied to studies of kidney development, adult healthy and diseased kidneys. As a result, they are driving paradigm shifts in molecular nephrology, offering new insights into complex biological processes such as kidney development and disease mechanisms. We will go through the representative, successful applications of single-cell multiomics analysis to nephrology below (Table 1).

**Table 1:** Single-cell multiomics sequencing kidney studies highlighted in this review

Condition	Modality	Sample	Cell numbers	References
Normal adult kidney	scRNA-seq scATAC-seq	Mouse kidney	43,636 cells 28,316 cells	Miao Z, et al. [48]
	scRNA-seq scATAC-seq	Human kidney	19,985 cells 27,034 cells	Muto Y, et al. [39]
	scRNA-seq scATAC-seq	Human kidney	50,768 cells	Ledru N, et al. [46]
Kidney development	scRNA-seq scATAC-seq	Mouse kidneys	7774 cells 10,765 cells	Hilliard S, et al. [50]
	scRNA-seq scATAC-seq	Mouse kidney Human kidney	109,477 cells 112,864 cells	Kim S, et al. [53]
	scRNA-seq scATAC-seq	Kidney organoid	56,865 cells	Yoshimura Y, et al. [51]
Acute kidney injury	scRNA-seq scATAC-seq	Mouse kidney Human kidney	157,000 cells 64,350 cells	Muto Y, et al. [55]
	scRNA-seq scATAC-seq	Mouse kidney	83,315 cells	Gerhardt LMS, et al. [56]
Acute kidney injury Chronic kidney disease	scRNA-seq scATAC-seq spatial transcriptomics	Human kidney	> 400,000 cells	Lake BB, et al. [58]
	scRNA-seq scATAC-seq spatial metabolomics	Human kidney	> 400,000 cells	Li H, et al. [60]
	scRNA-seq scATAC-seq	Human kidney	47,217 cells	Gisch DL, et al. [61]
Chronic kidney disease (hypertensive and diabetic kidney disease)	scRNA-seq scATAC-seq spatial transcriptomics	Human kidney	338,565 cells	Abedini A, et al. [59]
Diabetic kidney disease	scRNA-seq scATAC-seq	Human kidney	68,458 cells	Wilson PC, et al. [45]
	scATAC-seq Hi-C	Human kidney	38,847 cells	Eun M, et al. [62]
Autosomal dominant polycystic kidney disease	scRNA-seq scATAC-seq	Human kidney	102,710 cells 50,986 cells	Muto Y, et al. [44]
		Mouse kidney	125,434 cells	Muto Y, et al. [63]

#### Development and organoid

During organ development, the epigenetic landscape undergoes dynamic changes to coordinate the gene expressions required for cell-lineage specification [47]. Single-cell epigenetic analysis, such as scATAC-seq, provides critical insights into the dynamic cell-specific transcriptional regulation [28] underlying organ development. Miao et al. applied scATAC-seq and scRNA-seq to developing and adult mouse kidneys to elucidate cell-type-specific gene regulatory mechanisms, generating a list of key regulatory factors likely essential for differentiation into terminally differentiated renal epithelial cells [48]. Their analysis identified *Foxl1* gene as being specifically accessible and transcribed during podocyte commitment from nephron progenitor cells. Moreover, by integrating genome-wide association studies (GWAS) data with scATAC-seq, they demonstrated that disease-associated single nucleotide polymorphisms (SNPs) were enriched in cell-type- and developmental-stage-specific regulatory regions in mouse kidneys [48]. Their analysis underscores the utility of scATAC-seq in identifying target genes and cell types for GWAS variants, consistent with previous studies showing that SNPs are enriched in tissue-specific enhancer regions [49]. Characterizing rapidly transitioning biological states, such as progenitor populations during development, may benefit from simultaneous multimodal measurements rather than a combination of separated single-modality approaches. For example, the heterogeneity of *Six2* + nephron progenitor cells was better defined through joint profiling of single-cell transcriptomes and epigenomes compared to separately performed scRNA-seq and scATAC-seq [50]. This study highlighted the strength of simultaneous, paired single-cell multiomics analysis.

Yoshimura et al. applied paired single-cell multiomics analysis to the kidney organoid, demonstrating its relatively nonspecific genome-wide chromatin accessibility compared to adult kidneys [51], in line with the previous finding that the kidney organoids show developmental immaturity corresponding to the human fetal kidneys at the first trimester [52]. These findings suggested that paired single-cell multimodal analysis is useful for accurate cell-type annotation for kidney organoid. Motif enrichment analysis on their multiomics data indicated the critical role of HNF1B in the PT and TAL specification [51].

While much of our understanding of kidney development is derived from mouse studies, recent single-cell multiomics data from developing human kidneys have been integrated with mouse datasets [53]. Interspecies integration and subsequent comparative analysis have revealed species-specific and cell-type-specific regulatory programs. These data are anticipated to accelerate future research aimed at identifying causative genes of congenital kidney diseases and anomalies, and also improving our understanding of disease mechanisms by linking the fetal multiomics atlas to disease-associated GWAS data.

#### Adult healthy and diseased kidneys

We and others have employed single-cell multiomics profiling analysis for the human and mouse kidneys to construct multimodal atlases. Despite the small sample size, our aforementioned scRNA-seq and scATAC-seq datasets separately generated from five cortical kidney samples have efficiently dissected the heterogeneity of cortical epithelial cells [39]. We identified distinct subsets of thick ascending limb (TAL) cells, with the TAL1 subset characterized by *CLDN16* expression and the TAL2 subset marked by *CLDN10* expression separately in scRNA-seq and scATAC-seq data. These subpopulations exhibited differential transcription factor motif enrichment, implicating these transcription factors in driving TAL heterogeneity [39]. Notably, similar *Cldn10* + and *Cldn16* + TAL subtypes were also observed in the mouse scRNA-seq dataset [54]. We also applied our single-cell multiomics approach to human and mouse acute kidney injury (AKI) [55]. Through interspecies multiomics analysis, we identified key transcription factors, such as CREB5, that may be involved in both successful or failed epithelial repair in AKI [55]. Gerhardt et al. combined single-cell multiomics analysis and lineage-tracing of Ki67 + cycling population following AKI, describing a heterogeneous population of maladaptive PTCs and their epigenetic mechanism [56]. These studies suggested the strength of single-cell multiomics analyses for the characterization of low-abundance cell types. Another example of the successful application of single-cell multiomics to describe an underrepresented population was the characterization of erythropoietin-producing cells. Kragesteen et al. applied a single-cell multiomics approach to sorted erythropoietin-producing cells, identifying TCF21, CEBPD, and GATA6 as candidate master regulators, beyond the paradigm of HIF-2α–HIF-1β circuit in erythropoietin regulation [57].

Advances in single-cell technologies drive a decrease in the cost and development of sophisticated computational algorithms. These have fueled an exponential increase in the cell number included the datasets, allowing the generation of more comprehensive atlases. For instance, a multimodal atlas of human kidneys, encompassing single-cell transcriptomics, chromatin accessibility data, and spatial transcriptomics from 45 control and 48 diseased kidneys is generated [58]. This comprehensive atlas provided a high-resolution reference map for kidney cell types. The analysis of the dataset highlighted maladaptive cell states in PT and TAL cells associated with declines in eGFR. Abedini et al. generated a multiomics atlas from 81 human kidney samples, including scRNA-seq, scATAC-seq and spatial transcriptomics, capturing cells widely from both healthy and CKD kidneys [59]. They characterized the fibrotic niche, comprising heterogeneous cell populations such as fibroblasts, endothelial cells, immune cells, and maladaptive tubular cells, defining a fibrotic microenvironment (FME) gene signature. They have shown that the FME gene signature effectively classified kidney samples and predicted kidney prognosis [59].

Incorporating multiple omics layers into a single-cell atlas has further enhanced our understanding of kidney biology. For instance, spatial metabolomics combined with joint single-cell transcriptomics and epigenomics from 54 human kidney samples revealed that the same epithelial cell type exhibits distinct transcriptomic, epigenetic and metabolomic signatures depending on their anatomical location [60]. Gisch et al. integrated DNA methylation and genome-wide histone modifications profiling with scRNA-seq and scATAC-seq datasets in human kidneys, constructing a multimodal epigenome atlas [61]. Their study described a transcription factor network comprising ELF3, KLF6, and KLF10 that regulates the maladaptive PT cell state. Collectively, these studies have elucidated cell-type-specific transcriptional and epigenetic mechanisms driving CKD progression.

#### Diabetic kidney disease and polycystic kidney disease

Single-cell analyses have redefined cell states in specific kidney diseases, such as diabetic kidney disease (DKD). scATAC-seq analysis revealed a reprogramming of glucocorticoid responses in DKD kidneys [45]. Eun and Kim et al. employed a multimodal approach integrating scATAC-seq with Hi-C (a method for identifying genome-wide chromatin interactions) to investigate alterations in the epigenetic landscape of DKD. Their integrative analysis uncovered DKD-specific activation of BACH1, which was associated with maladaptive epithelial cell states [62].

Another successful application of multimodal single-cell analysis is in autosomal dominant polycystic kidney disease (ADPKD). We generated single-cell multiomics datasets from human ADPKD kidneys [44] and mouse PKD [63] models to redefine cystic cell types. Through analysis of these multimodal datasets, we identified a G-protein-coupled receptor GPRC5A as a novel cyst marker in both human and mouse kidneys. Our findings also revealed PKD-specific epigenetic alterations that drive GPRC5 A expression. Emerging lines of evidence suggest that GPRC5A is deregulated in many cancers, involved in several oncogenic pathways [64]. Hence, GPRC5A may have a role in PKD progression through modifying the survival and proliferation of cyst cells. These findings shed light on potential therapeutic targets and deepen our understanding of the molecular mechanisms underlying ADPKD.

### Future perspectives

Currently, single-cell studies of human kidneys are increasingly performed on diverse samples representing a broad range of genetic and environmental backgrounds as well as disease states. Alongside the rapid growth in the number of samples included in datasets, advance in spatial omics technologies are revolutionizing our multiomics approaches [65]. While most current spatial applications focus on transcriptomics, spatial omics is rapidly expanding to include epigenomics and proteomics. For example, spatial chromatin accessibility profiling methods have recently been applied to mouse and human brains [66], successfully defining the region-specific epigenetic landscapes in the brain. Epigenomic MERFISH, published in 2023, combines cleavage under targets and tagmentation (CUT-and-Tag) with multiplexed fluorescence in situ hybridization to spatially profile histone modifications [67]. This approach identifies active promoters and enhancers defined by histone modifications in a spatially-resolved manner. Another rapidly advancing technology in kidney research is spatial proteomics, which is based on either immunohistochemistry [68] or mass spectrometry [69, 70] to map protein distributions. Moreover, spatial proteogenomics, combining spatial proteomics and other omics modalities, has been applied to mouse and human livers, characterizing evolutionally conserved molecular pathways within hepatic cellular niches [71]. The future application of spatial epigenomics or proteogenomics in nephrology will significantly enhance our understanding of the molecular mechanisms underlying kidney development and diseases.

Furthermore, the ability of single-cell or multiomics analyses to characterize disease-specific cell states holds great potential for its future application to precision medicine. For example, Abedini et al. demonstrated that the fibrotic microenvironment gene signature could classify the samples of the kidneys and predict their prognosis [59]. Recent scRNA-seq analyses of urinary cells from DKD patients and healthy controls revealed the presence of nearly all kidney cell types in urine sediment, underscoring the potential of urine scRNA-seq as a non-invasive diagnostic tool [72]. Future applications of paired scATAC-seq and scRNA-seq on urine samples may allow comprehensive molecular monitoring of human disease progression and therapeutic response, offering valuable insights for both research and clinical practice, given the challenges associated with repeated renal biopsies due to their invasive nature.

Despite the substantial advantages of single-cell epigenetics and multiomics, several challenges persist. Cost and batch effects still remain significant obstacles in large-scale scRNA-seq and multiomics studies [73]. Sample multiplexing, now commercially available, offers a strategy to mitigate these issues, especially in scRNA-seq [73]. However, integrating datasets across multiple atlases generated by different laboratories, as well as large-scale inter-modality data integration, continues to pose challenges despite ongoing improvements in computational pipelines and benchmarking methodologies [8, 74]. Additionally, single-cell analyses often face limitations in analyzing rare cell populations and lowly expressed genes along with their epigenetic features, necessitating careful interpretation [73]. Nevertheless, continuous advancements in single-cell technologies are addressing these limitations and offering powerful tools to investigate the molecular mechanisms underlying kidney diseases.

## Conclusion

This review highlights the current advancements in single-cell sequencing analysis, with a focus on epigenetic profiling and emerging multiomics approaches in kidney research. Single-cell analyses have provided critical insights into cellular heterogeneity in human and mouse kidneys, offering new paradigms for molecular mechanism of kidney development and disease. The increasing availability of publicly accessible single-cell datasets as well as user-friendly bioinformatic tools continues to benefit the research community by fostering collaboration and innovation.

As single-cell multiomics technologies and computational tools continue to evolve, they will enhance the translation of discoveries from research to clinical nephrology. These advancements promise to drive advances in our understanding of kidney diseases, developing therapeutic targets, and implementing precision medicine approaches to improve patient care.
